# Environmental drivers of the leaf nitrogen and phosphorus stoichiometry characteristics of critically endangered *Acer catalpifolium*

**DOI:** 10.3389/fpls.2022.1052565

**Published:** 2022-12-16

**Authors:** Yuyang Zhang, Xiaoyu Cheng, Zhipeng Sha, Manuja U. Lekammudiyanse, Wenbao Ma, Buddhi Dayananda, Shuang Li, Ruiheng Lyu

**Affiliations:** ^1^ The National-Local Joint Engineering Laboratory of High Efficiency and Superior-Quality Cultivation and Fruit Deep Processing Technology on Characteristic Fruit Trees, College of Horticulture and Forestry Sciences, Tarim University, Alar, China; ^2^ Faculty of Morden Agricultural Engineering, Kunming University of Science and Technology, Kunming, China; ^3^ Coastal Marine Ecosystems Research Centre, Central Queensland University, Gladstone, QLD, Australia; ^4^ Ecological Restoration and Conservation of Forests and Wetlands Key Laboratory of Sichuan Province, Sichuan Academy of Forestry, Chengdu, China; ^5^ School of Agriculture and Food Sciences, The University of Queensland, Brisbane QLD, Australia; ^6^ State Key Laboratory of Environmental Criteria and Risk Assessment, Chinese Research Academy of Environmental Sciences, Beijing, China

**Keywords:** environmental factors, N/P ratio(L-N/P), leaf nitrogen content (LNC), leaf phosphorus content (LPC), Acer catalpifolium

## Abstract

*Acer catalpifolium* is a perennial deciduous broad-leaved woody plant, listed in the second-class protection program in China mainly distributed on the northwest edge of Chengdu plain. However, extensive anthropogenic disturbances and pollutants emissions (such as SO_2_, NH_3_ and NO_X_) in this area have created a heterogeneous habitat for this species and its impacts have not been systematically studied. In this study, we investigated the leaf nitrogen (N) and phosphorus (P) content of *A. catalpifolium* in the natural distribution areas, and a series of simulation experiments (e.g., various water and light supply regimes, different acid and N deposition levels, reintroduction management) were conducted to analyze responses of N and P stoichiometric characteristics to environmental changes. The results showed that leaf nitrogen content (LNC) was 14.49 ~ 25.44 mg g^-1^, leaf phosphorus content (LPC) was 1.29~3.81 mg g^-1^ and the N/P ratio of the leaf (L-N/P) was 4.87~13.93. As per the simulation experiments, LNC of *A. catalpifolium* is found to be relatively high at strong light conditions (80% of full light), high N deposition (100 and 150 kg N ha^-1^), low acidity rainwater, reintroduction to understory area or N fertilizer applications. A high level of LPC was found when applied with 80% of full light and moderate N deposition (100 kg N ha^-1^). L-N/P was high under severe shade (8% of full light), severe N deposition (200 kg N ha^-1^), and reintroduction to gap and undergrowth habitat; however, low L-N/P was observed at low acidity rainwater or P fertilizer application. The nutrient supply facilitates corresponding elements uptake, shade tends to induce P limitation and soil acidification shows N limitation. Our results provide theoretical guidance for field management and nutrient supply regimes for future protection, population rejuvenation of this species and provide guidelines for conservation and nutrient management strategies for the endangered species.

## Introduction

Ecological stoichiometry explains the relationship between the structure, function, and elements of the ecosystem in which plants themselves live (Elser and Hamilton, 2007; Sardans et al., 2012b). In plant leaf stoichiometry, N and P play an important role in the study of vegetation composition, ecosystem function, and nutrient constraints (Allen and Gillooly, 2009). The variation patterns of N and P concentrations and N/P ratio in leaves (L-N/P) and their influencing factors (biological and abiotic) have been widely reported (Reich and Oleksyn, 2004; Han et al., 2005; Ågren, 2008). Based on the previous findings, a series of hypotheses were constructed to explain the causes of leaf stoichiometry changes such as the biogeochemical cycle hypothesis, temperature-physiology hypothesis, plant growth rate hypothesis, and soil substrate age hypothesis (Chadwick et al., 1999; McGroddy et al., 2004; Ågren, 2008).

The biogeochemical hypothesis proposed that the storage capacity of the plant’s available nutrients in the soil is an important driving factor in the current status of leaf nutrients (McGroddy et al., 2004). The N from the environment (96.3 Tg N yr^-1^ of atmospheric N was deposited into the terrestrial ecosystems in 2016 (Ackerman et al., 2018)) into the system effectively improves the utilization and absorption of N by plants. A comprehensive analysis of 201 research reports found that simulated N deposition could increase the leaf nitrogen content (LNC) in terrestrial ecosystems, but leaf phosphorus concentration (LPC) was weak in response to N deposition (Sardans et al., 2017). Furthermore, previous studies suggested that N addition can significantly reduce the P concentration of plants aboveground due to massive imbalance of availability N and P which stimulates plant to take up more N but limit P uptake (Li et al., 2016b; Deng et al., 2017). In contrast, some studies have shown that N deposition increases the absorption of N and P by plants (Braun et al., 2009; Huang et al., 2012).

The temperature-physiology hypothesis suggests that plant metabolism is highly sensitive to temperature (Reich and Oleksyn, 2004). With the decrease in temperature, plant physiological domestication and adaptation increased LNC and LPC, while L-N/P increased accordingly (Reich and Oleksyn, 2004). Studies that used global databases have shown that LNC and LPC were higher in high latitudes than in low latitudes (Tian et al., 2017), and the change from relative P limitation to N limitation increased with the increase in latitude (Du et al., 2020). The soil substrate age hypothesis also predicts that the restriction of phosphorus from the Arctic (relatively young geologically) to tropical regions increases gradually, and is mainly limited by less weathered phosphate rock (Chadwick et al., 1999a).

The plant growth rate hypothesis has suggested that the relative growth rate of plants depends on the rate of protein synthesis (subject to phosphorus-rich ribosomal RNA) indicated plants relative growth rate mainly determined by the P concentration or P:N ratio of plants (Ågren, 2008; Peng et al., 2011). Under drought conditions, plants allocate more resources to growth and energy metabolism including the allocation of N, P and other elements to roots to facilitate water absorption (Gargallo-Garriga et al., 2014; Gargallo-Garriga et al., 2015). According to the results of literature integration analysis, drought can improve LNC on the whole but has no significant impact on LPC (Sardans et al., 2017). However, an arid environment can reduce the ability of plants to absorb and utilize nutrients and restrict plant growth (He and Dijkstra, 2014), which conforms to the plant growth rate hypothesis as a whole.

Experiments on field fertilization of plants show that the N:P of plants also have high internal stability (Demars and Edwards, 2007; Yu et al., 2010; Yu et al., 2011). However, N:P of a plant itself is not completely consistent with N:P in its habitat (water, air, and soil), which might be the reason for the plant’s ability in absorbing and storing nutrients to adapt to the habitat (Chapin et al., 1990; Chapin et al., 1993). This phenomenon is known as “resource supply-demand”, which is an asymmetric matching phenomenon of plants’ adaptation to their local environment (Güsewell, 2004). The stoichiometric characteristics of plants are usually defined by their limiting elements, which can be explained by “Liebig’s law of the minimum” (von Liebig, 1855). The demand for chemical elements in plants is limited by the element with the smallest supply (known as the limiting element), which has an important influence on the growth rate, population structure, and structural stability of plants (Vitousek et al., 2010). Hence, the study of plant restrictive elements is the premise to restore the population and judge the law of population development (Elser and Hamilton, 2007; Vitousek et al., 2010), N:P is an important indicator of plant nutrient restriction. Previous studies have suggested that low L-N/P (< 14) and high N:P (> 16) were driven by the N limitation and phosphorus limitation respectively, and the moderate L-N/P (14 < N:P < 16) can be the reason of both N and P limitation (Güsewell, 2004; Wassen et al., 2005).

Plant stoichiometry characteristics can be further affected by climate change scenarios (Vitousek et al., 2010). Due to anthropogenic activities (such as the burning of fossil fuels, transportation, and intensive agriculture), global reactive N emissions rise sharply, which can differ the N and P inputs in the terrestrial ecosystem (Vitousek et al., 2010). Moreover, an integrated analysis has shown that the significant changes in leaf stoichiometry are related to the increased CO_2_ concentration, drought conditions, and P enrichment (Sardans et al., 2017).

*Acer catalpifolium* Rehd. belongs to the family Sapindaceae and is a large broad-leaved deciduous tree with a tall and straight trunk, which has been listed in the “wild plants with extremely small populations” (WPESP) rescue and protection plan due to the critically endangered status in China (Zhang et al., 2020). Previous field investigation stated the narrow distribution of this species (mainly in the rain zone of west China) and serious obstacle to the regeneration of natural population (Zhang et al., 2018). This distribution area is affected by anthropogenic emissions with large N deposition and frequent acid rains which have profoundly affected the *A. catalpifolium* habitat (Du et al., 2020; Zhang et al., 2021a; Zhang et al., 2021b), however, it is still unclear how these emissions change the N and P content in the soil and at what level these changes can affect the growth of *A. catalpifolium*. We conducted field investigations combined with a series of simulation experiments to analyze the driving factors of L-N/P as well as the response to different environmental change scenarios.

## Materials and methods

### Field investigation

In November 2017, mature and healthy *A. catalpifolium* leaves were collected from the natural populations from Leibo, Yibin, Dujiangyan, Ya’an, Emei mountain, and Pingwu regions in Sichuan province. A total of 22 sampling sites were selected from the above locations, and nine samples were taken from artificial planting *A. catalpifolium* forest (10 years old) in Emei mountain. Three healthy plants were selected from each sampling site, and 10 healthy and intact leaves were collected from each plant. In addition, soil samples were collected (n=6) at each corresponding sampling site. Part of the collected soil samples were stored in a -20 °C refrigerator before measuring the available N content, and the rest was air-dried to measure other properties (including soil pH, organic matter, total N, available P and K). The longitude and latitude, altitude, tree height, and diameter at breast height (DBH) of the sampling sites were recorded (detailed procedure refer to Zhang et al. (2018)).

### Simulation experiment

The simulation experiments were carried out in Beijing (different water and light simulation experiments) and Tangchang Town of Sichuan Province (simulated acid and N deposition experiments). The seedlings of *A. catalpifolium* used in simulation experiments were grown in a seedbed that contained Burozem and bark sawdust humus. After one month of cultivation, the seedlings with similar growth characteristics were transplanted into pots (one plant per pot) and prepared for experimental treatments. The size of the pot was 35cm × 45cm, the soil used for potting was same as that in the seedbed, both Burozem and bark sawdust humus, soil organic matter 2.52%; total N 0.14%; total P 0.24%; total K 0.47%; available P 27.21 mg kg^-1^; available K 134.68 mg kg^-1^.

### Simulated different water and light conditions experiment

After transplantation, seedlings were placed in three light conditions (L1, L2, and L3 represented 80%, 50% (using single layer black nylon mesh net), and 8% (using double layer black nylon mesh net) of total light, respectively) which were manipulated by the different layers of black mesh nylon nets, and four water treatments (W1, W2, W3, and W4 represented 35%, 55%, 75% and 95% water holding capacity, respectively). Each treatment was replicated six times (Zhang et al., 2019).

### Simulated acid deposition experiment

The acid deposition experiment was conducted from 1^st^ May 2017 to 31^st^ October 2018 and was set up as two acid deposition patterns (leaf and soil application) × two acid rain types (sulfuric acid dominant acid rain, and nitric acid balanced acid rain) × three acidity gradients (pH 2.5, 3.5 and 4.5). The control (CK) also included and only received filtered water. The sulfuric acid dominant acid rain applied to leaf and soil was expressed as SL (SL2.5, SL3.5, and SL4.5) and SS (SS2.5, SS3.5 and SS4.5) respectively, nitric acid balanced acid rain applied to leaf and soil are expressed as NL (NL2.5, NL3.5, and NL4.5) and NS (NS2.5, NS3.5 and NS4.5) respectively (Zhang et al., 2021b). Each treatment was replicated six times. The leaves were collected from each treatment at end of the experiment.

### Simulated N deposition experiment

The simulated N deposition experiment was conducted at the same time with an acid deposition experiment. Five N deposition levels (i.e., 30, 60, 100, 150, and 200 kg N ha^-1^ y^-1^represented by N3, N6, N10, N15, and N20, respectively) and CK (only received filtered water) were included in this experiment. The N addition was conducted every 15 days, with 20 additions for the entire duration of the pot experiment. Different NH_4_NO_3_ aqueous solutions (prepared according to the treatment concentrations) of 300 ml were added to corresponding treatment pots and a similar volume of water was added to the CK. Each treatment was replicated six times. Daily field management routines such as irrigation and weeding were kept the same with the simulated acid deposition experiments. The leaves samples were collected from each treatment at end of the experiment.

### Wild reintroduction experiment

#### Various light environments in forest

*Acer catalpifolium* with the same growth features was selected and transplanted to its natural habitat (located in Western Asia Alpine Botanical Garden, Institute of Plants, Chinese Academy of Sciences) in November 2017. Five A*. catalpifolium* were planted at a spacing of 2 m × 2 m under each light condition (including unshaded land, forest edge, gap and understory) in the forest (n=5).

### Soil fertilization experiment

In this experiment, we included six soil amendments, which were N fertilizer (urea, 46% N, 1 kg per plant), P fertilizer (calcium superphosphate, 12% P_2_O_5_, 4 kg per plant), NP fertilizer (1 kg urea per plant plus 4 kg calcium superphosphate per plant), vermicompost (10 kg per plant), charcoal (10 kg per plant). *A. catalpifolium* planted at the spacing of 2 m × 2 m, soil amendments were applied to different treatments in two times (the first is planting time and the second is in April 2018). Daily weeding, pest control, and irrigation were carried out to ensure the normal growth of *A. catalpifolium*.

### Chemical analysis of soil and plant samples

Fully expanded and disease-free leaves of *A. catalpifolium* were collected from field investigation, simulation experiments and wild reintroduction experiments and leave samples were oven-dried with 105 °C for 30 mins and then 85 °C for 72 h until a constant weight resulted. Leaf samples were ground and digested by H_2_SO_4_-H_2_O_2_ solution and determined the N and P content using an AA3 continuous flow analyzer. Soil properties such as soil pH, soil organic matter, soil total N content, soil available nitrogen ( ${\text{NO}}_{3}^{-}$ -N and ${\text{NH}}_{4}^{+}$ -N), phosphorus and potassium were measured using the determination methods described by Sha et al. (2020).

### Data analysis

In order to clarify the effects of explanatory variables on nutrient concentration and L-N/P characteristics of *A. catalpifolium*, explanatory variables were divided into soil factors, geographical factors and species factors, and the strong colinearity and irrelevant variables were eliminated through multiple linear regression analysis combined with stepwise regression analysis model.

The IBM SPSS 22 (IBM Corp, 2013) was used to analyze the significant difference in leaf N and P content as well as L-N/P under different treatments (p<0.05). Multivariate regression analysis were performed using R Program 4.1.1 (R Core Team, 2021). Figures were developed by using Origin 9.0 and R 4.1.1.

## Results

### Leaf N and P stoichiometric characteristics of *Acer catalpifolium* and its drivers

**Figure 1:**
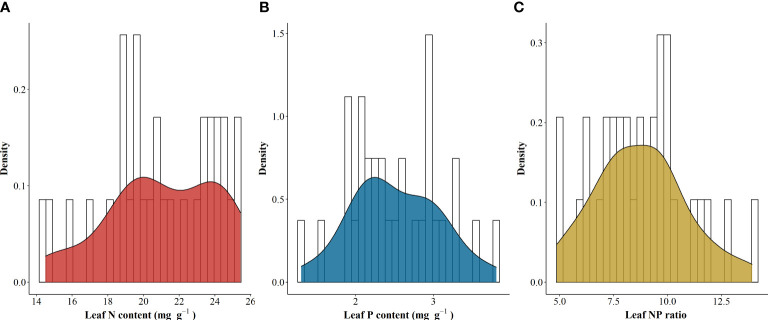
Leaf N and P stoichiometry traits of *Acer catalpifolium*. **(A)** leaf N content, **(B)** leaf P content and **(C)** leaf N/P ratio.

The LNC and LPC of *A. catalpifolium* were 20.02 mg g^-1^ (14.49-25.44), 2.27 mg g^-1^ (1.29-3.81) (**Figures 1A, B**), and the mean L-N/P of *A. catalpifolium* were 8.75 (4.87-13.93) (**Figure 1C**).

**Figure 2:**
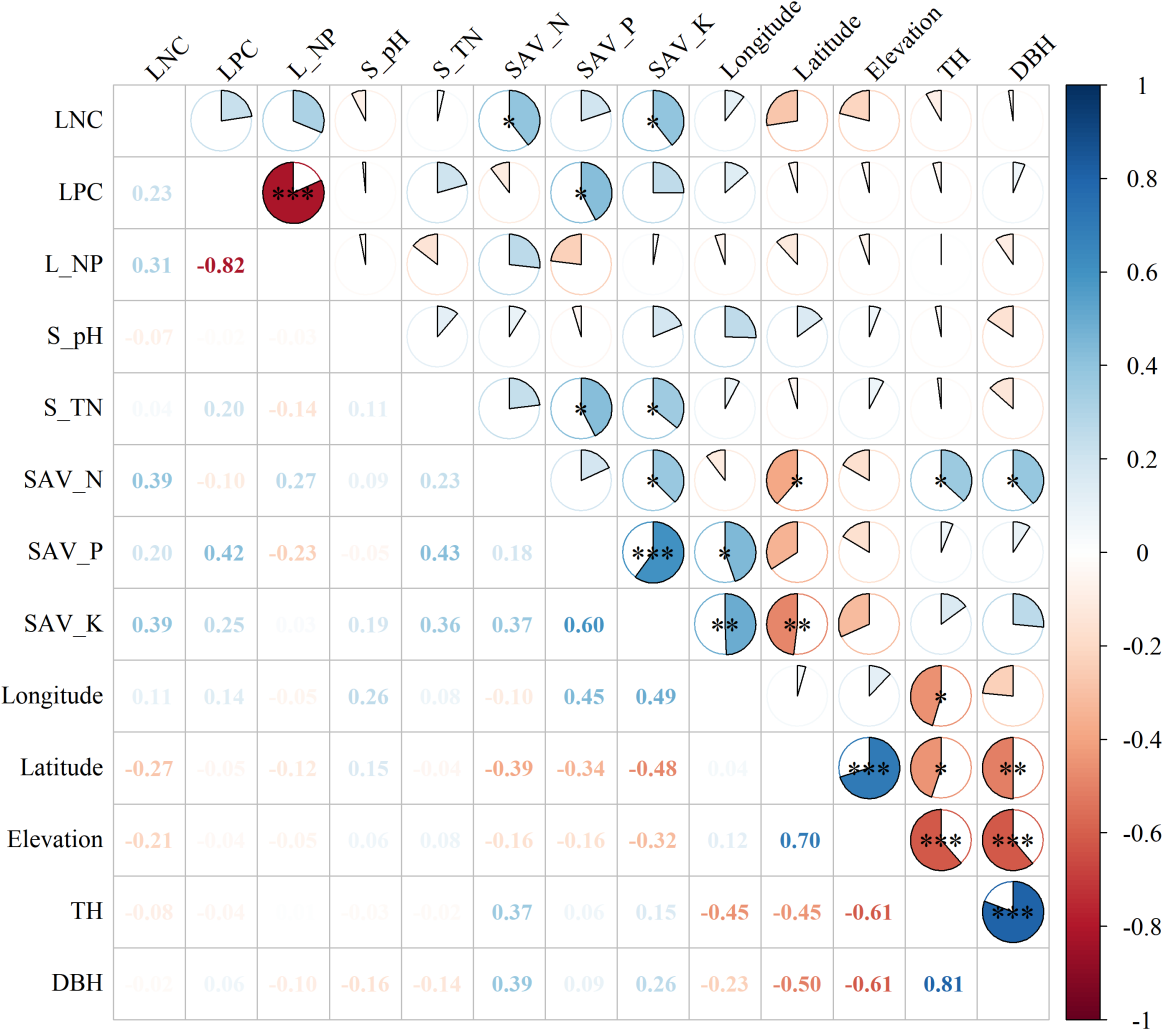
Pearson’s correlation matrix of leaf nutrition and environmental explanatory variables. Note: S_pH, S_TN, SAV_N, SAV_P, SAV_K, TH, and DBH represent soil pH, soil total nitrogen, soil available nitrogen, soil available phosphorus, soil available potassium, tree height, and DBH, respectively. Asterisk (*) indicated significant correlation with *, ** and *** indicating significance levels at 0.05 0.01 and 0.001, respectively.

LNC was significantly correlated with soil available N (SAV_N), potassium (SAV_K) (**Figure 2**), the correlation coefficients were 0.39 and 0.39. The LPC was significantly correlated with soil available P (SAV_P) and the correlation coefficient was 0.42. Tree height (TH) was negatively correlated with longtitude, latitude and elevation, and positively correlated with SAV_N, the correlation coefficients were -0.45, -0.45, -0.61 and 0.37 respectively.

The changes in LNC were mainly controlled by soil pH, total N, available N, available K, altitude and DBH, and the overall explanation rate of N concentration of *A. catalpifolium* was 31% (**Table 1**). The LPC was only related to soil available P, and its explanation rate for LPC change was 15%. In terms of L-N/P, consistent with Pearson correlation analysis and redundancy analysis, the L-N/P of *A. catalpifolium* was mainly affected by soil available N and P, and the explanation rate of L-N/P variation was 19%.

Table 1Multivariate regression analysis of N/P in the foliage of *Acer catalpifolium*.

**Table:** 

Variables		LNC		LPC		L-N/P	
		Estimate	*P*	Estimate	*P*	Estimate	*P*
	(Intercept)	75.82	0.21	16.53	0.22	-14.93	0.76
**Soil**	S_pH	-0.61	0.32	0.08	0.53	-0.56	0.26
	S_TN	-0.34	0.17	0.01	0.88	-0.17	0.38
	SAV_N	0.42	0.02*	-0.04	0.26	0.25	0.06
	SAV_P	0.00	0.74	0.01	0.05	-0.02	0.10
	SAV_K	0.01	0.05	0.00	0.54	0.00	0.49
**Geography**	Longitude	-0.64	0.28	-0.16	0.22	0.30	0.52
	Latitude	0.69	0.36	0.09	0.59	-0.15	0.80
	Elevation	-0.01	0.06	0.00	0.90	-0.00	0.69
**Species**	TH	-0.14	0.24	-0.03	0.21	0.09	0.30
	DBH	-0.07	0.23	0.02	0.21	-0.11	0.04*
**F**		2.05		0.92		1.09	
**R^2^**		0.25		0.02		0.03	
***P***		0.08		0.52		0.41

**Table:** 

Variables		LNC		LPC		L-N/P	
		Estimate	*P*	Estimate	*P*	Estimate	*P*
	(Intercept)	27.96	0.00***	2.35	0.00***	7.43	0.00***
**Soil**	S_pH	-0.76	0.14	—	—	—	—
	S_TN	-0.30	0.16	—	—	—	—
	SAV_N	0.39	0.01**	—	—	0.17	0.04*
	SAV_P	—	—	0.01	0.02*	-0.01	0.06
	SAV_K	0.01	0.04*	—	—	—	—
**Geography**	Longitude	—	—	—	—	—	—
	Latitude	—	—	—	—	—	—
	Elevation	-0.00	0.11	—	—	—	—
**Species**	TH	—	—	—	—	—	—
	DBH	-0.12	0.01**	—	—	—	—
**F value**		3.25		6.35		3.50	
**R^2^**		0.31		0.15		0.19	
***P***		0.02*		0.02*		0.04*	

Asterisk (*) indicated significant impact, with *, ** and *** indicating significance levels at 0.05 0.01 and 0.001, respectively.

**Figure 3:**
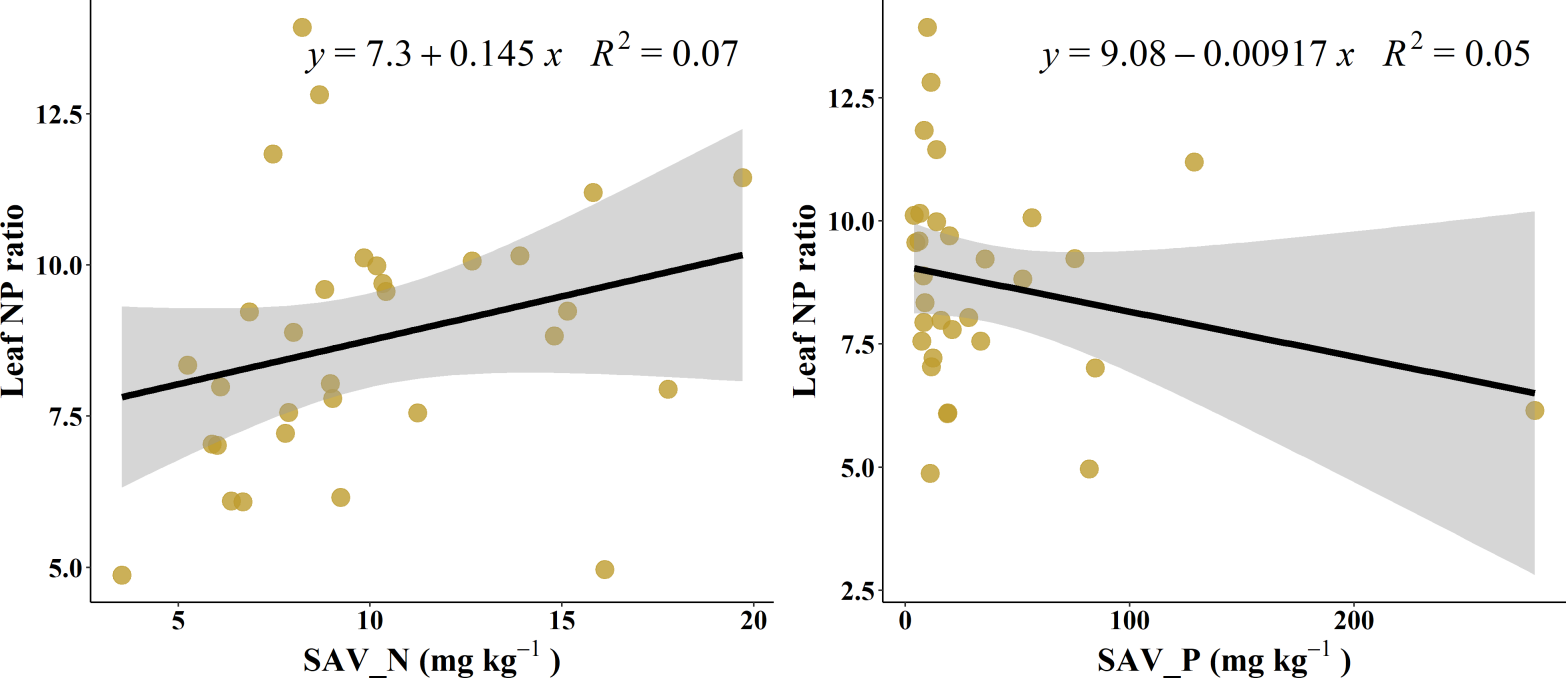
Correlation of leaf NP ratio with soil available nutrients.

At the single factor level, the explanation rate of soil available N to the change of L-N/P was only 7.1% (**Figure 3A**), and that of available P was 5.3% (**Figure 3B**).

### Effects of water and light on N and P stoichiometric characteristics

**Figure 4:**
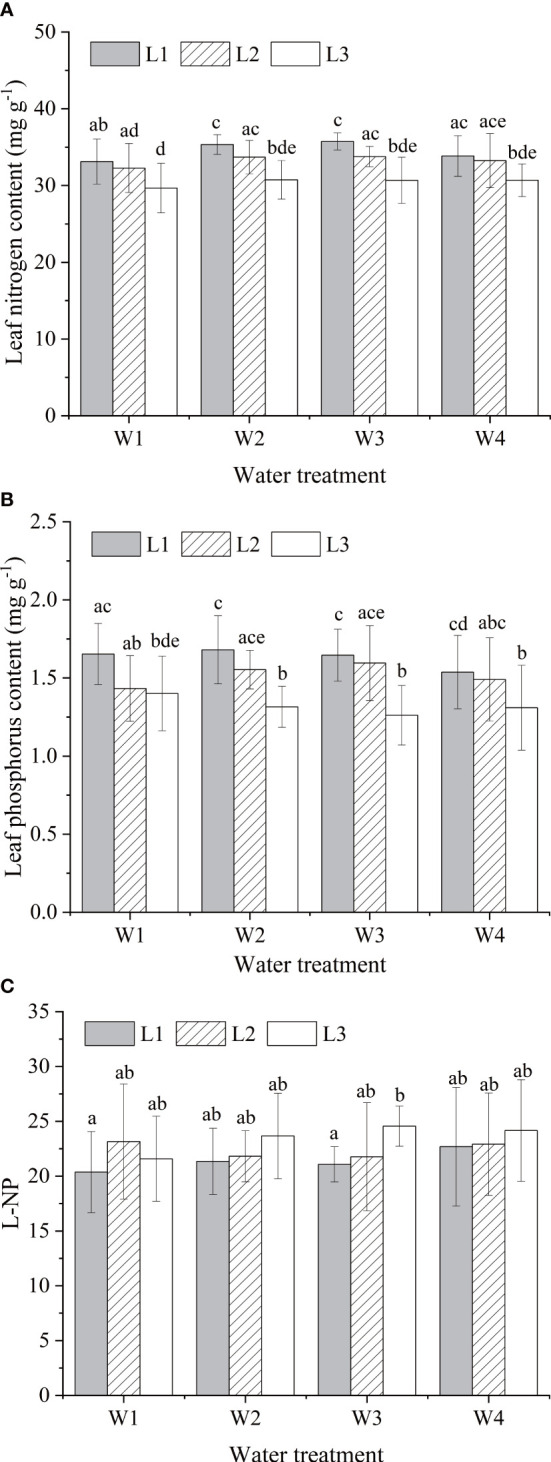
Effects of water and light on characteristics of leaf N and P of *Acer catalpifolium*. **(A)** leaf N content, **(B)** leaf P content and **(C)** leaf N/P ratio.

**Figure 5:**
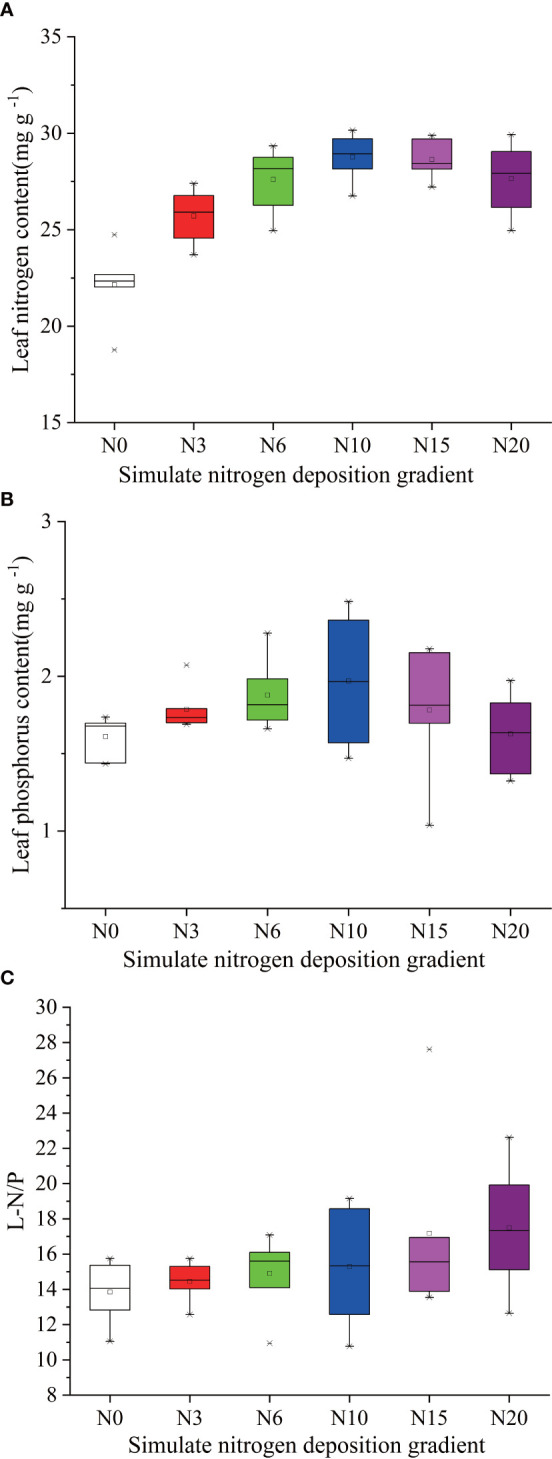
Effects of simulated N deposition on characteristics of leaf N and P of *Acer catalpifolium*. **(A)** leaf N content, **(B)** leaf P content and **(C)** leaf N/P ratio.

Different water and light supply regimes have changed foliar N and P composistion of *A. catalpifolium*. The LNC was high in L1 light condition, of which L1W2 and L1W3 treatments were significantly higher than those of L2W1 and L3 light treatments (p<0.05) (**Figure 4A**). The LPC was also higher under L1 light, but under the same light condition, there was no significant difference in LPC among different water treatments (p>0.05) (**Figure 4B**). The L-N/P in L3W3 treatment was significantly higher than that in W1 and W3 treatment under L1 light (p<0.05) (**Figure 5C**).

### Effects of simulated acid deposition on stoichiometric characteristics of N and P in *Acer catalpifolium* leaves

Significant variations in foliar N and P content under different acid deposition patterns were observed. Leaf acid spraying did not significantly change the LNC of *A. catalpifolium* with CK. The LNC under NL4.5 treatment was significantly higher than that under NL2.5 treatment (p<0.05) (**Table 2**). The LNC under NS3.5 and NS4.5 treatments were significantly higher than that under NL2.5 treatment (p<0.05). The LPC of NS2.5 and SS2.5 was significantly higher than that of CK, NL2.5, and NL2.5 (p<0.05). The L-N/P of NS2.5 and SS2.5 was significantly lower than that of NL3.5 and NL4.5 (p<0.05).

**Table 2:** Effects of simulated acid deposition on characteristics of leaf N and P of *Acer catalpifolium*.

Added method	Treatments	Acidity	LNC(mg g^-1^)	LPC(mg g^-1^)	L-N/P
**—**	CK	—	22.15 ± 1.93bcd	1.61 ± 0.14bc	13.85 ± 1.78ab
**Leaf spray**	NL	2.5	20.46 ± 2.96b	1.42 ± 0.29b	14.79 ± 2.89ab
		3.5	23.03 ± 3.75ab	1.48 ± 0.37bd	16.95 ± 7.11a
		4.5	24.72 ± 3.71ac	1.59 ± 0.24bc	15.82 ± 2.76ac
	SL	2.5	22.08 ± 3.35bc	1.44 ± 0.23b	15.47 ± 2.61acd
		3.5	22.52 ± 3.21ab	1.57 ± 0.18bc	14.59 ± 3.02ab
		4.5	21.72 ± 3.85bc	1.51 ± 0.28bc	14.50 ± 1.85ab
**Soil added**	NS	2.5	25.68 ± 1.72ad	2.41 ± 0.85a	11.44 ± 2.81b
		3.5	25.01 ± 3.35ac	1.97 ± 0.44acd	13.04 ± 2.59bc
		4.5	25.88 ± 2.36a	2.01 ± 0.29ac	13.01 ± 1.24bc
	SS	2.5	23.53 ± 3.36ab	2.22 ± 0.60a	11.33 ± 3.56b
		3.5	24.36 ± 2.37ac	2.14 ± 0.49a	11.79 ± 2.15b
		4.5	24.82 ± 3.21ac	2.16 ± 0.62ac	12.06 ± 2.83bd

### Effects of simulated nitrogen deposition on stoichiometric characteristics of N and P in *Acer catalpifolium* leaves

Simulated different N deposition levels have altered foliar nutrients status, significantly increased the LNC of *A. catalpifolium* (p<0.05) and the LNC increased with the increase of N addition (**Figure 5A**). The changes of LPC in the leaf of *A. catalpifolium* under simulated N deposition were similar to those of LNC (**Figure 5B**). The LPC under N10 treatment was significantly higher than CK (p<0.05) (**Figure 5B**). The L-N/P under N20 treatment was significantly higher than CK (p<0.05) (**Figure 5C**).

### Effect of different canopy density on leaf N and P stoichiometric characteristics of *Acer catalpifolium* leaves

**Figure 6:**
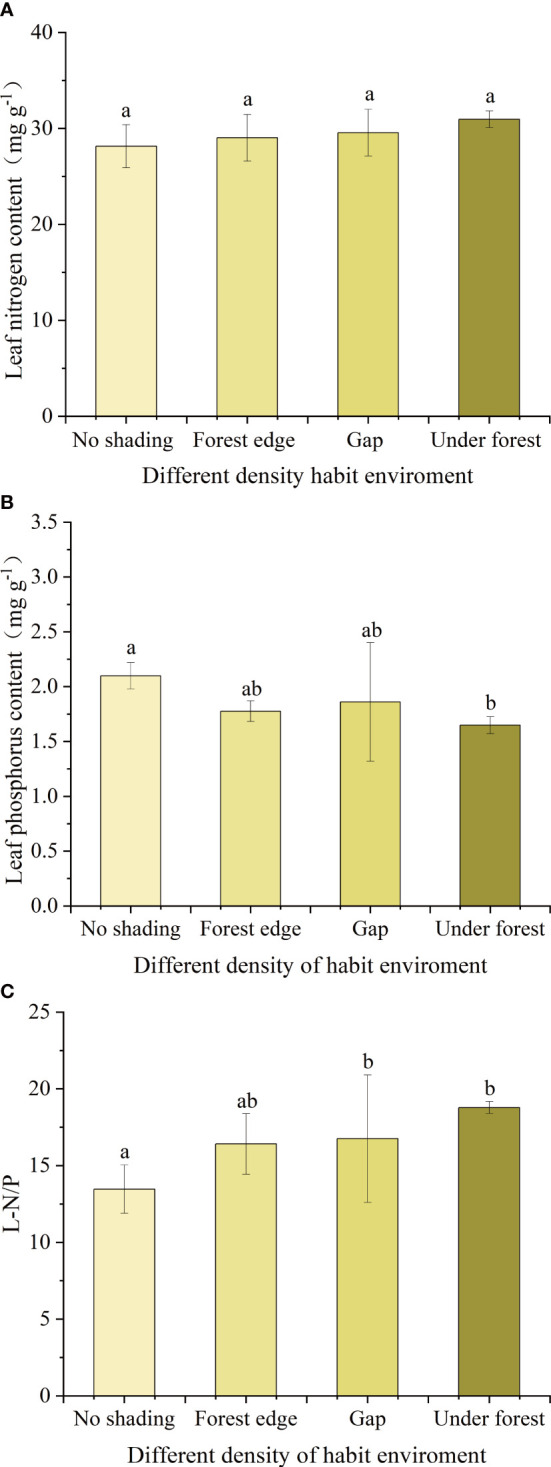
Effects of different light conditions on characteristics of leaf N and P of *Acer catalpifolium*. **(A)** leaf N content, **(B)** leaf P content and **(C)** leaf N/P ratio.

Under different canopy density habitats, LNC of leaves of *A. catalpifolium* was higher in the light environment of the understory, but there was no significant difference among treatments (p>0.05) (**Figure 6A**). On the contrary, the LPC of *A. catalpifolium* was lower in understory light and significantly lower than that of no shading treatment (p<0.05) (**Figure 6B**). The L-N/P under gap and understory light was higher than that that of no shading (p<0.05) (**Figure 6C**).

### Effects of soil amendment on leaf N and P stoichiometric characteristics of *Acer catalpifolium*

**Figure 7:**
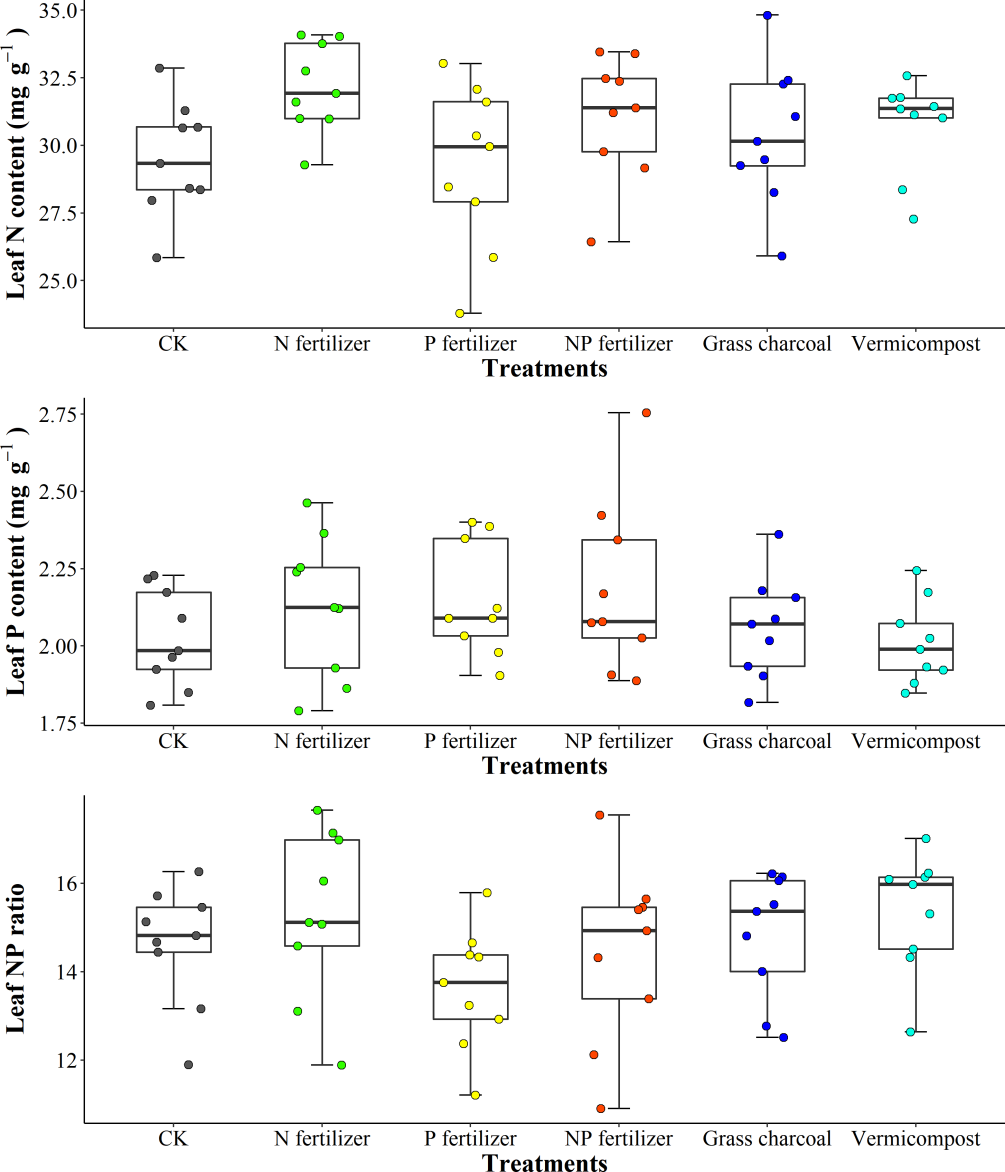
Effects of different soil fertility improvement measures on characteristics of leaf N and P of *Acer catalpifolium*.

The amendments application changed soil nutrients pool. N fertilizer application increased the LNC of *A. catalpifolium* which was significantly higher than that of CK and P fertilizer (p<0.05) (**Figure 7A**). Different soil amendment applications had little effect on the LPC of *A. catalpifolium* (**Figure 7B**). The L-N/P ratio of leaves treated with P fertilizer was lower compared to N fertilizer and vermicompost (p<0.05) (**Figure 7C**).

## Discussion

The results from field investigation, simulation experiments and wild reintroduction experiments provided a quantitative perspective of the effect of environmental changes in leaf N and P stoichiometric characteristics of *Acer catalpifolium*. The LNC and LPC of *A. catalpifolium* in this study were found higher than that of the data synthesis study which indicated the average LNC and LPC of woody plants (based on the global plant leaf N and P stoichiometry dataset) are 18.22 g kg^-1^ and 1.10 g kg^-1^, respectively (Tian et al., 2017). Stoichiometric studies of 753 species in China also showed relatively low LNC (18.6 g kg^-1^) and LPC (1.21 g kg^-1^), but the L-N/P (14.4) was higher compared to this study (Han et al., 2005). L-N/P is an important indicator of plant nutrient restriction. Previous studies have pointed out that when L-N/P < 14, nutrients are N limited, l-N/P > 16 is P limited, and when 14 < L-N/P < 16, it is common N and P limited (Güsewell, 2004; Wassen et al., 2005). The range of L-N/P of *A. catalpifolium* was 4.87 ~ 13.93 in the rain zone of west China, indicating that there was a strong N limitation in the distribution area of *A. catalpifolium*. The L-N/P is affected by factors such as geography, climate and soil, the temperature-physiology hypothesis holds that plant metabolism is more sensitive to temperature, and plant physiological domestication and adaptation increase plant LNC and LPC, while L-N/P increases with the decrease of temperature (Reich and Oleksyn, 2004). Results from a global database confirm that plant LNC and LPC are higher at high latitudes (Tian et al., 2017), and change from relative P limit to N limit (i.e., L-N/P increase) with increasing latitudes (Du et al., 2020).

*Acer catalpifolium* had a small geographical distribution gradient (the mid-latitude variation range in this survey was only 29.36°~32.24°). Therefore, the contribution of geographical explanation variables to N and P stoichiometry of *A. catalpifolium* was weak according to stepwise regression analysis. The soil substrate age hypothesis predicts that the restriction of P increases gradually from geologically young Arctic to tropical regions and is mainly limited by less weathered phosphate rock (Chadwick et al., 1999), and the biogeochemical hypothesis pointed out the storage capacity of plants available nutrients in the soil largely reflect the current status of leaf nutrients (McGroddy et al., 2004). In this study, soil available N (ammonium N + nitrate N) and soil available P (Olsen-P) contributed the most to the changes of N and P stoichiometry of *A. catalpifolium*, both of which reached significant levels. Among species factors, stepwise regression analysis showed that there was a negative correlation between DBH of *A. catalpifolium* and LNC, which was inconsistent with previous studies (Liu et al., 2020). In this survey, the DBH of *A. catalpifolium* had a large variation range (The DBH ranges from 8 cm to 54 cm), and the correlation between DBH and LNC used different tree ages which may be responsible for the difference from previous findings.

Light and water are key factors that limit plant growth, plant growth rate hypothesis holds that the relative growth rate of plants depends on the rate of protein synthesis (restricted by P-rich ribosomal RNA), in other words, the relative growth rate of plants is positively correlated with plant P concentration and P/N (Ågren, 2008; Peng et al., 2011). The relative growth rate of *A. catalpifolium* decreased with the decrease of light conditions (Zhang et al., 2019), and its corresponding LNC and LPC also decreased (especially under W2 and W3 moisture conditions), while L-N/P showed an increasing trend. This is consistent with previous studies (Wang et al., 2013); The plants in the water-light test had significantly higher LNC but lower LPC than those in the *A. catalpifolium* distribution area, and the higher LNC was mainly due to the high frequency of water recharge in the greenhouse test that facilitated soil N mineralization (Dijkstra et al., 2016), while the soil used in this study was typical calcareous fluvo-aquic soil in north China, and the reaction between soil P and calcium limited the effectiveness of P thus making the LPC relatively (Graf, 1980). Moreover, differences in NP stoichiometry at different growth stages (saplings were studied in the water and light trials) likewise made an important contribution to the differences in leaf nutrients between the two sites.

In the acid deposition study, the LNC of *A. catalpifolium* was similar to that of plants in the distribution area but differed significantly from the Beijing water and light test (**Figure 4**), consistent with previous studies on *Acer mono* at different study sites also found large differences in LNC concentrations (27.91 ± 0.58 mg g^-1^ in Beijing and 12.99 ± 1.30 mg g^-1^ in Shanxi) but small differences in LPC (Song et al., 2019). Acid rain acting on plant leaves alters cell permeability thereby leaching important elements from the leaves thus affecting leaf morphology and physiological functions (Zeng et al., 2005; Liu et al., 2018). LNC was significantly lower in the NL2.5 treatment but similar changes were not observed under sulfuric acid dominant acid rain treatment, further demonstrating that HNO_3_ has a stronger effect on leaf related to stronger oxidative stress on physiological activity than H_2_SO_4_. In addition, the LNC increased significantly under the low acidity treatment and was slightly higher than CK, which is consistent with previous studies (Hu et al., 2019), The presence of abundant available N in acid rain at low acidity is beneficial for leaf uptake, counteracting the negative effects of acid rain or even further enhancing leaf physiological activities such as increasing LNC, chlorophyll and photosynthetic rate (Dong et al., 2017a; Dong et al., 2017b). Differing from leaf acid spraying, soil acid addition directly enriched the available N pool in soil and aided in nutrient uptake of *A. catalpifolium*. Additionally, the soil acidification due to the continuous input of exogenous H^+^ and could improve P acquisition by plants, just like the simulated forest soil acidification experiment, soil P bioeffectiveness was found to be increased by 15-30% (Ohno and Amirbahman, 2010), and similar patterns were observed in other agricultural fields and water bodies (Kopáček et al., 2015; Wang et al., 2017). In the simulated acid rain experiment, the CK of *A. catalpifolium* showed co-limitation of N and P (**Table 2**). Leaf acid spray did not significantly change L-N/P in our study, but the LPC elevation was high under the soil acid addition treatment (especially high acidity treatment) and the leaves shifted from NP co-limitation to N limitation. These results were consistent with Hu et al. (2019) ‘s study on the influence of simulated acid rain on the stoichiometry of *Camellia sinensis*. However, it is worth noting that this experiment was only conducted for one year of continuous acid addition and the results may change in long-term acid addition. With the long-term aggravation of soil acidification, the mobility of Al, Mn and other elements can further increase and make toxic conditions in the plant that limit the plant growth and P absorption efficiency (Yang et al., 2008).

Environmental N (atmospheric N deposition) into terrestrial ecosystems effectively increases N uptake by plants. Simulated N deposition (N addition) was found to increase LNC by 22% (from 225 observations) in terrestrial ecosystem plants, and LNC by 30% approximately (from128 observations) in temperate forests (Sardans et al. (2017)). Similarly, the LNC of catalpa maple was found to be increased by 16-30% under the influence of simulated deposition and the response of LPC to N deposition was lower relative to LNC (Sardans et al., 2017). Some studies targeting the aboveground parts of plants have also concluded that N addition significantly reduces P concentration (Li et al., 2016a; Deng et al., 2017), it has also been suggested that plants can respond to increased N resources by increasing P uptake or by enhancing P resorption capacity (Sardans et al., 2012a). The weak response of LPC to simulated N deposition found in this study, L-N/P increased significantly by 15% and *A. catalpifolium* shifted from a co-limitation of N and P to a limitation of P (especially N15 and N20 treatments). Similarly, a previous study based on data integration has found that N addition can increase L-N/P by 10-27% (Sardans et al., 2017).

In the wild reintroduction experiment, the LNC responded to the N fertilizer, P fertilizer and N, P fertilizer treatments by the same mechanism as the simulated N deposition plant response. We found that the LPC under N and P treatments increased by 6% and 8% compared to CK, respectively, which is lower than the previous findings on LPC (Sardans et al., 2017) and the difference may be related to the frequency of fertilizer addition (only twice) and treatment cycle (Li et al., 2016b; Li et al., 2019). In this study of field habitat selection, the change of L-N/P of *A. catalpifolium* was also small, and L-N/P was reduced by P application, which was consistent with previous research results (Sardans et al., 2017). However, we observed non-significant increase in L-N/P between N and NP treatments (**Figure 7C**). A study pointed out that long-term N addition had a more significant change in L-N/P (Fujita et al., 2010). Among the fertilization treatments, peat soil and vermicompost mainly improved soil structure and promoted root growth, and N and P concentration and N and P ratio in leaves of *A. catalpifolium* showed little response to the addition of two substrates (**Figure 7C**). However, further follow-up studies are needed to clarify the long-term effects of substrate addition.

In the habitat experiment with different canopy densities, the essence is to explore the effects of different light environments on the nutrients of *A. catalpifolium*. The LNC of *A. catalpifolium* showed a weak response to different canopy density treatments, but the LPC of *A. catalpifolium* growing under the forest was relatively low, resulting in a relatively high L-N/P. This is consistent with the results of the water-light experiment and is consistent with the plant growth rate hypothesis, namely, the increase of P uptake by plants with increasing light intensity is positively correlated with the P/N (Ågren, 2008; Peng et al., 2011).

## Conclusion

The stoichiometric characteristics of N and P in leaves of *A. catalpifolium* in field population and applied with different treatments were studied. The results showed that the LNC, LPC and L-N/P in the field population were 14.49~25.44 mg g^-1^, 1.29~3.81 mg g^-1^, and 4.87~13.93 respectively. The N/P in the wild population was affected by soil available N and P. The LNC and LPC were higher under high light treatment. Under simulated N deposition, LNC and LPC of *A. catalpifolium* were the highest applied with N10 treatment, while L-N/P was the highest for N20 treatment. Under simulated acid deposition, LNC and LPC were higher in soil application with high acidity treatment and L-N/P was high in leaf application. Applied to different canopy closure habitats, LNC of *A. catalpifolium* was higher, LPC was lower in the understory, and L-N/P was higher in gap and understory habitats. The LNC of N fertilizer treatment was significantly increased compared with the CK, while the LPC of *A. catalpifolium* increased slightly under substrate and fertilization, and the L-N/P of *A. catalpifolium* was lower under P fertilization. These results provide solid guidance for field management and nutrient supply regimes for the future protection and population rejuvenation of this species. Moreover, this provides guidelines to develop and implement a suitable management strategy such as conservation and nutrient management, and translocation plan to manage the regeneration of populations and help prevent the loss of remaining populations.

## Data availability statement

The raw data supporting the conclusions of this article will be made available by the authors, without undue reservation.

## Author contributions

ZS and RL conceived and designed the study and provided suggestions and comments for the manuscript. YZ and XC collected and analyzed the data and wrote the manuscript. SL and YZ collected the samples. BD, ML, and RL revised the manuscript. All authors read and approved the manuscript.
